# Impact of a Service Provider Incentive Payment Scheme on Quality of Reproductive and Child-health Services in Egypt

**DOI:** 10.3329/jhpn.v28i3.5556

**Published:** 2010-06

**Authors:** Dale Huntington, Hassan H.M. Zaky, Sherine Shawky, Faten Abdel Fattah, Eman El-Hadary

**Affiliations:** ^1^ Reproductive Health and Research Department, World Health Organization, Geneva, Switzerland; ^2^ Social Research Center, American University in Cairo, Egypt; ^3^ Faculty of Economics and Political Sciences, Cairo University, Cairo, Egypt

**Keywords:** Performance payments, Child Health, Reproductive health, Impact studies, Quality of care, Case-control studies, Health services, Egypt

## Abstract

A case-control, quasi-experimental study was designed (post-test only) to investigate the effect of a performance-based incentive payment scheme on behaviours of public-sector service providers in delivering a basic package of maternal and child-health services in Egyptian primary healthcare units. The results showed significant improvements in the quality of family-planning, antenatal care, and child-care services as reported by women seen in clinics where the incentive payment scheme was in operation as measured by various indicators, including both technical and inter-personal communication content. An analysis of characteristics of the service providers and clients found no significant or meaningful differences between the study groups, and the facilities of both the study groups were essentially the same. Some findings are suggestive of other influences on behaviours of the service providers not captured by the data-collection instruments of the study. Subsequent to this study, the payment scheme has been rolled out to other districts in Egypt.

## INTRODUCTION

There is an extensive literature on different types of payment methods for healthcare providers and the effects of incentives on organizations and individuals in the healthcare system (1, 2). The evidence on incentive payments based upon specific performance criteria is generally mixed, though suggestive of several positive effects on the quality of care and, cost-containment measures. However, the findings in many settings are difficult to interpret as the introduction of payment reforms is often accompanied with other changes in the service-delivery setting that affect the quality of care.

Incentives are known to elicit complex responses from physicians, inducing changes in the number of hours worked for the number of beneficiaries seen per hour, the location of their work, and the type of service provided to a patient (3, 4). The introduction of incentive payments linked to the productivity of the service provider can lead to over-valuing certain procedures or services, producing inefficiencies and unnecessary care. For example, payments made to clients, medical personnel, and outreach workers who motivate and refer clients for family planning have been a concern for national family-planning programmes (5). In general, experience with the incentive payment schemes shows that they can lead to multiple impacts on behaviours of service provider—both intended and unintended—and must be carefully monitored (6–8).

There are also other risks in the use of incentive payments. For example, performance-based incentive payment schemes increase the level of administrative costs because these require data on the number and type of services provided and have been criticized for these hidden costs (9, 10). Thus, the success of an incentive payment system will depend upon the efficient operations of the financial and management systems that underpin the payments (11) and careful selection of the performance measures.

Despite these other well-known difficulties and risks, payments of salary (either in part or whole) that are linked to performance measures are attractive policy options as a means to improving the quality of service and gaining efficiencies.

### Setting of study

Beginning in the middle of the 1990s, the Government of Egypt began to openly grapple with solutions to pressing problems that the piecemeal approach to reform used previously had failed to resolve. There were significant equity problems in access to services, by both income and geographical groupings, and public spending on health was regressive (12). The organization of the health sector and its management was burdened with a cumbersome mix of centralized and decentralized functions. Financing of the health sector was fragmented and uncoordinated, prohibiting effective risk-pooling and encouraging predatory behaviours among service providers. The delivery system was characterized by substantial excess capacity and under-use of sub-standard quality facilities. There were both surpluses and imbalances of medical personnel, with an over-supply of specialists, under-supply of primary care physicians, and absolute shortage of nurses.

The 1997 Health Sector Reform Strategy Paper responded to these challenges by setting a long-term vision of universal coverage with basic health services for all citizens (13). The pilot phase (1998–2004) of the Egyptian Health Sector Reform Program (HSRP) focused on primary healthcare in a Family Health Project that modelled several basic reform principles, including creating a Family Health Fund (a social insurance scheme) to reduce out-of-pocket expenditure for a Basic Benefit Package that includes reproductive health services (family planning, maternal and newborn care) (14).

At the close of the HSRP pilot phase, several important initiatives had been introduced (e.g. basic benefit package) while other reforms had not yet been evaluated. Among the later was a set of reforms targeting payments to healthcare providers that were developed in part a response to long overdue increases in salaries in the public sector and also as a means for improving the quality of care. Several types of payment reforms were being explored, including contracting mechanisms (both contracting-in and contracting-out for services) using incentive payments linked to performance measures.

### Description of incentive payment scheme

The Family Health Fund works through the District Provider Organizations to contract with public and private providers to offer the Basic Benefit Package to the covered populations. Initially, the fund was designed to disburse payments on a per-capita basis system but this was soon put on hold as its implementation required substantial modifications to the existing procedures and policy that could not be achieved during the pilot phase. As a consequence, the Family Health Fund shifted towards the use of salary supplements in the form of incentive payments to encourage facilities to maintain certain operating standards and performance targets.

Under this scheme, the incentive payments may reach up to 275% times the total base salaries of all personnel working in the Primary Health Center Unit (PHCU). The payments are metred according to performance measured against a set of standardized indicators and rating criteria (14). The indicators include both curative and preventive services, in addition to the quality of care-related indicators, e.g. completeness of medical records, satisfaction of patients, waiting-time, etc. Minimum target levels are set based on the national and Governorate programme goals. The sources of data for the incentive payment scheme include both routinely-collected service statistics and datasets generated through clinic supervision visits made by the District Provider Organizations (which include exit-interviews with patients and facility-inspection checklists). No special data-collection activities or new indicators are used by the incentive payment scheme.

The performance indicators are weighted differentially to encourage service providers to give more attention to indicators of priority programmes, e.g. family planning and immunization. A numeric score which forms the basis for calculating the actual amount of the incentive to be disbursed to each service provider according to a weighting system that differentiates between three categories of staff in each facility: healthcare providers (physicians and nurses), administrative staff, and clerks.

The incentive payment scheme was being phased into selected District Provider Organizations and PHCUs at the close of the HSRP pilot phase in 2004. In settings where the incentive payment scheme was not being introduced, all service providers of the Ministry of Health and Population (MoHP) were receiving the same amount of salary supplement but as a top-off of their regular salary, i.e. not based on any performance assessment. This phased-in approach to implementing the incentive payment scheme created a natural experimental setting for studying the effects of the incentive payment compared to a flat-rate salary supplement.

This study aimed at testing the hypothesis that providers who receive an incentive payment will provide better-quality services and be more responsive to the healthcare needs of their clients than providers who receive a salary supplement that is not linked to performance.

## MATERIALS AND METHODS

### Study design and sampling procedures

The study used a quasi-experimental post-test only comparison group design that tested the following hypothesis: Providers who receive the incentive payment will provide better-quality reproductive health services and be more responsive to the clients’ needs for reproductive healthcare than providers who received a salary supplement not linked to performance.

Results on indicators relating to the performance of service providers and patient outcomes in primary healthcare units where providers received incentive payments were compared with results from primary healthcare units where providers did not receive incentive payments but did receive an equivalent amount as salary top-off. The study included indicators used by the incentive payment scheme and other measures of satisfaction of patients and quality of clinic services.

Two (Menoufia and Suhag) of the five Governorates that piloted the incentive payment scheme were purposively selected for this study based upon considerations of location (Lower and Upper Egypt) and length of time that the payment scheme had been in place (e.g. more than 2 years). The two Governorates were selected because they each were working with District Provider Organizations (a feature of the health reform programme).

Within each Governorate, a single district recognized as being the most active and engaged with the implementation of the HSRP was purposively selected for the study sample: El-Maragha health district in Suhag and Quesna health district in Menoufia. This was done to ensure the likelihood of finding the impact of the incentive payment scheme and to hold constant system-wide improvements that could influence the quality of care provided in the study sites.

In each of the selected districts, all the PHCUs offer the same basic benefit package of services and have taken part in all other elements of the HSRP to equal measure (e.g. all the PHCUs are fully accredited and are similarly constructed, with comparable types of materials and medical equipment) but only some have been using the incentive payment scheme. The incentive payment clinics were somewhat better finished than the non-incentive payment PHCUs, e.g. a larger waiting-room and newer benches in the waiting-area. However, all the PHCUs in both the study groups were rated equal by the quality accreditation scheme of the MoHP. Thus, the most salient difference between the two types of PCHU in the study sample is the incentive payment scheme. The former units represent the frame for the intervention units of the study while the latter units are the frame for the comparison group. In total, four PHCUs were selected for the intervention group, and four PHCUs were selected for the comparison group.

Within each of the selected PHCUs in each district, all the clinic physicians were interviewed using semi-structured, qualitative discussion guides. The managers of the District Provider Organization were also interviewed. The study sampling frame included all consenting women of reproductive age (15–49 years) at the selected PHCUs. These interviews were conducted upon the patient's exit from the clinic using a standardized quantitative questionnaire administered by a trained interviewer.

### Sample characteristics

Eighty-one healthcare providers—52 in Menoufia Governorate and 29 in Suhag Governorate—were interviewed. Of them, 46 were males and 35 were females. Other than gender, there were no significant differences in the professional or personal characteristics between the physicians in the incentive payment scheme sites and the comparison group, although physicians in the incentive payment sites were somewhat more likely to be younger and have a higher educational degree. Importantly, there were no significant differences in the training programme experiences between the service providers in each study group.

In total, 2,414 women were interviewed. Approximately, an equal number (600) was interviewed in each study group by Governorate (i.e. 600 cases, 600 comparison group in each Governorate). Overall, there were no significant differences between the two study groups by age, educational level, working status, or education and working status of their husbands. There were significant differences (p<0.05) between the two study groups by age at first pregnancy, number of living children, number of living sons and girls, and previous history of miscarriage and by the wealth quintiles. For example, women in the incentive group were more likely to have had their first pregnancy at a later age, have fewer children, suffered fewer deaths of children, and be of slightly higher economic status (i.e. one group difference as shown by quintile analysis of wealth) than women in the comparison group.

The volume of services seen in the study clinics during the 11-month period (January-November 2006) before data collection was reviewed to detect any differences in case-load. The average number of reproductive health patients seen per month per PHCU was approximately twice as high in the non-incentive payment scheme units (335 patients per month per clinic versus 184 patients per month per clinic, respectively). However, the number of consultations not related to reproductive health was higher in the incentive payment sites, resulting in no significant difference in the total number of consultations for all reasons between the two study groups. Anecdotal information collected during the study suggests that the lower reproductive health case-load in the incentive group can be attributed to the influence of a large general hospital in one Governorate located nearby the incentive payment scheme PHCU. The consultation fee at the hospital is one-third the cost of care at the incentive payment scheme PHCU. There are no other fees charged (for medicines or laboratory analyses), and clients are seen by a specialist doctor. A general practitioner sees clients in the incentive scheme units, and the fee includes only 50% of the prescribed medicine cost.

The primary reason for visiting the PHCU during the data-collection period of the study is shown in Table 1. Childcare was the most frequently-used service in both incentive and non-incentive scheme units, as around half of the service users in each study group visited for childcare, followed by antenatal care and family-planning service. The incentive payment scheme units were somewhat more likely to provide family-planning and antenatal care services than the non-incentive payment scheme units, although the difference was not significant.

**Table 1.** Reasons for seeking healthcare in the incentive and non-incentive primary healthcare units of the two Egyptian Governorates, Provider Incentive Payment Study, Egypt, 2007

Type of service	Menoufia*		Suhag*		Total†	
	Incentive scheme (n=601)	Non-incentive scheme (n=607)	Incentive scheme (n=606)	Non-incentive scheme (n=600)	Incentive scheme (n=1,207)	Non-incentive scheme (n=1,207)
Family planning	14.6	4.0	6.3	7.3	10.4	5.6
Antenatal care	16.1	8.6	14.0	10.5	15.1	9.5
Gynaecology	0.7	1.5	1.2	1.0	0.9	1.2
Childcare	55.7	68.0	45.7	43.5	50.7	55.8
Others**	12.8	18.0	32.8	37.7	22.9	27.8

*Menoufia incentive units: Begeram and Mastai; Menoufia non-incentive units: Kafr Bani Gheriyan and Ta Shubr; Suhag incentive units: Nage Helal and Nage Tayee; and Suhag non-incentive units: Banaweet and Nage Tammam;

†The differences in proportions between incentive and non-incentive schemes in Menoufia and total reached statistical significance (p<0.05);

**Others: healthcare services other than reproductive and child-healthcare services

## RESULTS

The incentive payment scheme had a clear impact on the performance of family-planning care providers, with significant differences observed with regard to better history-taking, less laboratory investigations, more follow-up visits, and more information about the available family-planning methods (Table 2). In addition, the family-planning clients in the incentive payment scheme units were significantly more likely to report having assisted in the choice of the contraceptive method than the clients in the non-incentive payment sites. This is a critically-important indicator of the quality of care and has been associated with sustained use of family planning in other research (15–17). Some important differences between the Governorates emerged in the disaggregated results. In Menoufia, family-planning clients seen in clinics where the incentive scheme was operating were more likely to have a complete history taken, asked about the date of their last menstrual cycle, previous contraceptive-use, and the history of past illness, among other indicators (Table 2). The experience in Suhag Governorate was inconclusive about the effects of the incentive payment scheme on the same indicators. This could be attributed to the lower case-load of family-planning clients in the Suhag study sites due to the presence of nearby hospitals (mentioned previously).

**Table 2.** Family-planning care offered to women in the incentive and non-incentive primary healthcare units of the two Egyptian Governorates, Provider Incentive Payment Study, Egypt, 2007

Parameter	Menoufia		Suhag		Total	
	Incentive scheme (n=88)	Non-incentive scheme (n=20)	Incentive scheme (n=28)	Non-incentive scheme (n=41)	Incentive scheme (n=116)	Non-incentive scheme (n=61)
History-taking						
Name	83.0	65.0	71.4	70.7	80.2	68.9
Age	65.9	55.0	67.9	68.3	66.4	63.9
Parity	64.8	50.0	67.9	58.5	65.5	55.7
Date of last menses*	69.3	40.0	71.4	61.0	69.8	54.1
Previous contraception†	61.4	30.0	75.0	61.0	64.7	50.8
History of past illness†	63.6	20.0	67.9	65.9	64.7	50.8
Medical examination						
Palpation of breast	5.7	0.0	17.9	22.0	8.6	14.8
Vaginal examination	9.1	5.0	25.0	9.8	12.9	8.2
Investigation						
Laboratory investigation*	3.4	35.0	35.7	22.0	11.2	26.2
Family-planning follow-up**					
Asked to return*	75.0	50.0	69.6	63.4	73.9	59.0

*The differences in proportions between incentive and non-incentive schemes in Menoufia and Total reached statistical significance (p<0.05);

†The differences in proportions between incentive and non-incentive schemes in Menoufia reached statistical significance (p<0.05);

**The total number of cases who were asked about family-planning follow-up in the Suhag incentive scheme PHCUs was 23 as five cases left the PHCU without using contraception method. The total number of incentive scheme beneficiaries was 111 women

The incentive payment scheme had several positive effects on the quality of child healthcare services, which was the most common reason for having visited the PHCU during the study. In total, 1,286 women attended the selected units to obtain care for a child during the study (not shown in Table 3). The age of the children seeking healthcare ranged from less than one month to 15 years and was not different between the two study groups, or by Governorate. Forty-five percent of the children were suffering from an upper respiratory tract infection (any child with fever suffering from cough and/or sore throat and/or difficulty in breathing). Eruptive infectious diseases were the second common cause for seeking care (fever with skin eruption), followed by diarrhoea (with or without fever and with no symptoms of upper respiratory tract infection).

**Table 3.** Type of childcare services provided in incentive and non-incentive primary healthcare units

Type of care	Menoufia		Suhag		Total	
	Incentive scheme (n=328)	Non-incentive scheme (n=413)	Incentive scheme (n=273)	Non-incentive scheme (n=259)	Incentive scheme (n=601)	Non-incentive scheme (n=672)
Prescribed treatment	92.4	99.5	91.9	96.1	92.2	98.2
Received injection	8.8	a 2.2	9.5	14.3	9.2	6.8
Follow-up	52.1 (n=303)	23.7 (n=411)	58.2 (n=251)	41.7 (n=249)	54.9 (n=554)	30.7 (n=660)
Child given medicine	11.9	5.6	29.9	16.1	20.0	9.5
Explained medicine	96.4	88.6	95.2	78.3	95.8	84.7
Women knew medicine-use	99.0	90.3	99.6	82.3	99.3	87.3

All differences are significant at p<0.05

The results presented in Table 3 show an apparent tendency towards better childcare practices in the incentive scheme PHCUs than the non-incentive scheme PHCUs. The care providers in the incentive scheme PHCUs were more likely to request for follow-up than those in the non-incentive scheme PHCUs and are less likely to prescribe medicines but when they did prescribe they were more likely to administer injections immediately. Carefully monitoring a child through follow-up coupled with the less-frequent use of medication can lead to a reduction in the practice of over-prescription. Mothers of the children in the incentive scheme sites were more likely to report having received clear instructions on how to care for the sick child than those in the non-incentive scheme sites.

These results are shown in both the Governorates and in the study's overall results.

In general, there were clear results, suggesting several positive impacts of the incentive scheme payments on antenatal care (Table 4). The antenatal care patients in the incentive scheme were significantly more likely to have a complete medical history taken, undergo more complete examination, and laboratory tests made than in the non-incentive scheme clinics. The exception was tetanus toxoid (TT), which was actually lower in the incentive payment scheme clinics in Menoufia than in the non-incentive payment scheme sites. This may be because the care providers in the non-incentive payment scheme were less likely to retrieve or consult the medical files of the patients (e.g. to check if a TT immunization was needed) than care providers in the incentive payment scheme group (results not shown in Table 4).

**Table 4.** Antenatal care offered to women in incentive and non-incentive primary healthcare units of two Egyptian Governorates

Parameter	Menoufia		Suhag		Total	
	Incentive scheme (n=97)	Non-incentive scheme (n=52)	Incentive scheme (n=85)	Non-incentive scheme (n=63)	Incentive scheme (n=182)	Non-incentive scheme (n=115)
History-taking						
Name	96.9	96.2	94.1	87.3	95.6	91.3
Age	96.9	94.2	96.5	90.5	96.7	92.2
Parity	92.8	b 80.8	91.8	81.0	92.3	a 80.9
Date of last menses	95.9	90.4	94.1	93.7	95.1	92.2
History of past illness	89.7	a 57.7	83.5	81.0	86.8	a 70.4
Examination						
Weight	92.8	88.5	96.5	88.9	94.5	88.7
Blood pressure	94.8	b 84.6	97.6	90.5	96.2	a 87.8
Foetal heart rate	12.4	3.8	61.2	b 41.3	35.2	24.3
Investigations						
Blood test	84.5	a 63.5	92.9	87.3	88.5	a 76.5
Urine analysis	72.2	a 28.8	96.5	92.1	83.5	a 63.5
Management						
Tetanus toxoid	59.8	a 80.8	45.9	46.0	53.3	61.7
Iron	54.6	65.4	52.9	61.9	53.8	63.5
Vitamins	38.1	25.0	56.5	55.6	46.7	41.7
Treatment	17.5	13.5	35.3	46.0	25.8	31.3

Note: statistical significant for differences by Governorate and combined are denoted by a: p<0.01;

b: 0.01<p<0.05 for indicators

The incentive scheme had an impact on behaviours of the doctors who were significantly less likely than their colleagues in the non-incentive scheme clinics to prescribe unnecessary medicines, more likely to take a full history (and to record it in a medical file), and more likely to ask their patients if they had any questions and encourage them to return for a follow-up.

The results of the interviews with the physicians in the PHCU and the district healthcare officers (District Provider Organization) revealed mixed feedback on the design and functioning of the incentive payment scheme. The main highlights of these views were as follows:

Too many indicators were used in the calculation of the incentive, and the level of details was overly micro in focus.

The indicators were established by national-level decision-makers without consulting local administration. This caused many problems during the implementation as both district-and facility-level care providers needed time to understand the construction of the payment scheme and the rationale for using the selected indicators.

Calculations of percentage of the incentive and reasons of its reduction were not clear to most physicians, reflecting an overly-detailed approach.

The delays in receiving incentives created an atmosphere of distrust and uncertainty.

The phased introduction of the scheme caused some confusion with the overall management of the district—different approaches should be adopted for launching the scheme.

## DISCUSSION

The incentive scheme improved those things which scored points in the scheme, such as record-keeping (which was significantly better in the incentivised clinics) and the non-clinical aspects of behaviours of doctors, such as the clarity of their communication and listening to their clients. Although improvements in the quality of care were associated with the introduction of the incentive scheme, the study also showed overall low levels of quality in both the study groups, e.g. women reporting clear communication with the care providers. Sustained attention is required for the continual improvement of quality of service, and greater efforts are needed for consumer education that will empower patients with skills to gather the type of information needed to ensure compliance and the sustained use of contraceptive methods.

Contextual differences also emerged in the findings of the study, indicating the limitations of an incentive payment scheme in overcoming external influences on behaviours of the care providers or how management and supervision may also exert an influence on behaviours of the care providers. For example, for many key quality-of-care indicators, the clinics in Suhag Governorate scored higher than those in Menoufia, regardless of whether they were or not in the incentive scheme. These differences between the Governorates are suggestive of other factors influencing behaviours of the care providers beyond the incentive payments. The study did not explore the possibility of how the incentive payment scheme may have changed the overall configuration of services, perhaps leading to a ‘crowding-out’ of other, non-incentivized services which can occur as the providers focus on services that are linked to the incentive payments and not on other, non-incentivised services.

Although the doctors and managers were supportive of the incentive payment scheme, they complained that they were not fully consulted during its design. Consequently, they felt that the scheme was too complicated and that the weights given to different indicators were changed too often.

The results from the early introduction of the incentive payment scheme in Egypt are highly suggestive that the care providers do respond to incentives that are carefully integrated into a well-known and established quality of the care-monitoring system. Although the introduction of the new payment scheme was cumbersome for the district officials and caused confusion for the physicians working in the scheme's sites, overall, the experience has been positive. The incentive payment scheme continues to be used in Egypt, expanding into other Governorates as an important element of the national health-sector reform programme.
